# Supplementary material for: Environmental microbiome diversity and stability is a barrier to antimicrobial resistance gene accumulation
Source: Commun Biol. 2024 Jun 8;7:706. doi: 10.1038/s42003-024-06338-8 (PMC11162449; doi:10.1038/s42003-024-06338-8)
Supplement: Supplementary file 2 — Supplementary information [file 42003_2024_6338_MOESM2_ESM.pdf]

Supplementary information for:

Environmental microbiome diversity and stability is a barrier to antimicrobial resistance gene accumulation

Uli Klümper[1,*], Giulia Gionchetta[2,*], Elisa Catão[3,4,*], Xavier Bellanger[3], Irina Dielacher[5], Alan Xavier Elena[1], Peiju Fang[1], Sonia Galazka[6], Agata Goryluk-Salmonowicz[7], David Kneis[1], Uchechi Okoroafor[8], Elena Radu[5,9], Mateusz Szadziul[7], Edina Szekeres[10], Adela Teban-Man[10], Cristian Coman[10], Norbert Kreuzinger[5], Magdalena Popowska[7], Julia Vierheilig[5,11], Fiona Walsh[8], Markus Woegerbauer[6], Helmut Bürgmann[2,+], Christophe Merlin[3,+], Thomas Ulrich Berendonk[1,+,#]

[1] Technische Universität Dresden, Institute for Hydrobiology, Dresden, Germany

[2] Eawag, Swiss Federal Institute of Aquatic Science and Technology, Department of Surface Waters – Research and Management, Kastanienbaum, Switzerland

[3] Université de Lorraine, CNRS, LCPME, Villers-lès-Nancy, France

[4] Université de Toulon, MAPIEM, Toulon, France

[5] TU Wien, Institute of Water Quality and Resource Management, Vienna, Austria

[6] AGES – Austrian Agency for Health and Food Safety, Department for Integrative Risk Assessment, Division for Risk Assessment, Data and Statistics, Vienna, Austria

[7] University of Warsaw, Faculty of Biology, Institute of Microbiology, Department of Bacterial Physiology, Warsaw, Poland

[8] Maynooth University, Department of Biology, Kathleen Lonsdale Institute for Human Health, Maynooth, Co. Kildare, Ireland

[9] Romanian Academy of Science, Institute of Virology Stefan S. Nicolau, Bucharest, Romania

[10] NIRDBS, Institute of Biological Research Cluj-Napoca, Cluj-Napoca, Romania

[11] Interuniversity Cooperation Centre Water & Health, Vienna, Austria

* These authors contributed equally

+ These authors jointly supervised this work

# Corresponding author

Corresponding author address:

Prof. Thomas Berendonk (ORCID: 0000-0002-9301-1803)

Technische Universität Dresden

Institute of Hydrobiology

Zellescher Weg 40

01217 Dresden

Germany

Tel. +49 351 463-34956

Mail thomas.berendonk@tu-dresden.de

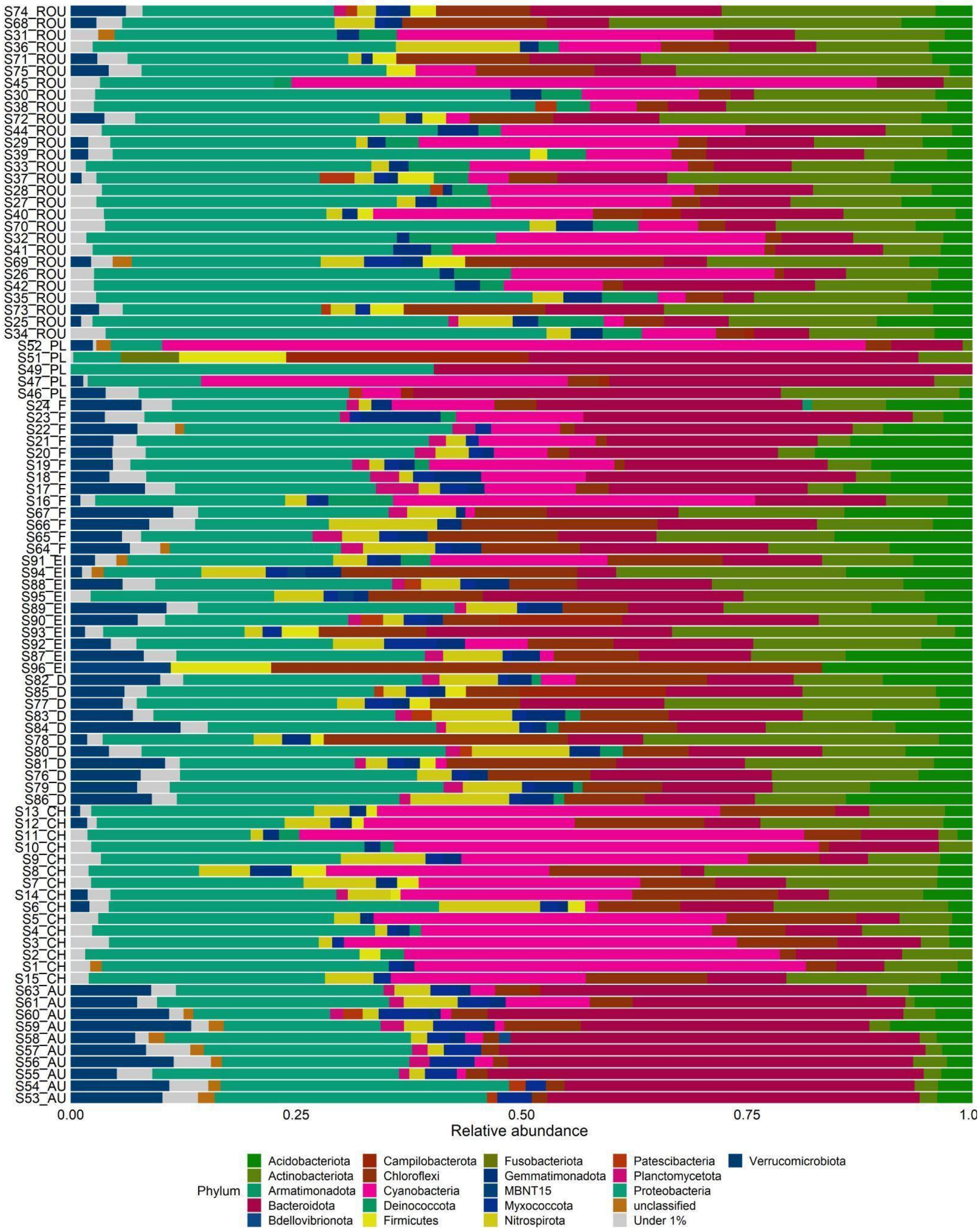

Supplementary Figure 1. Barplot of phylum diversity in all rivers sampled across 7 countries. For each sample, the phyla with less than 1% relative abundance were clustered as "Under 1%" to help with visualization.

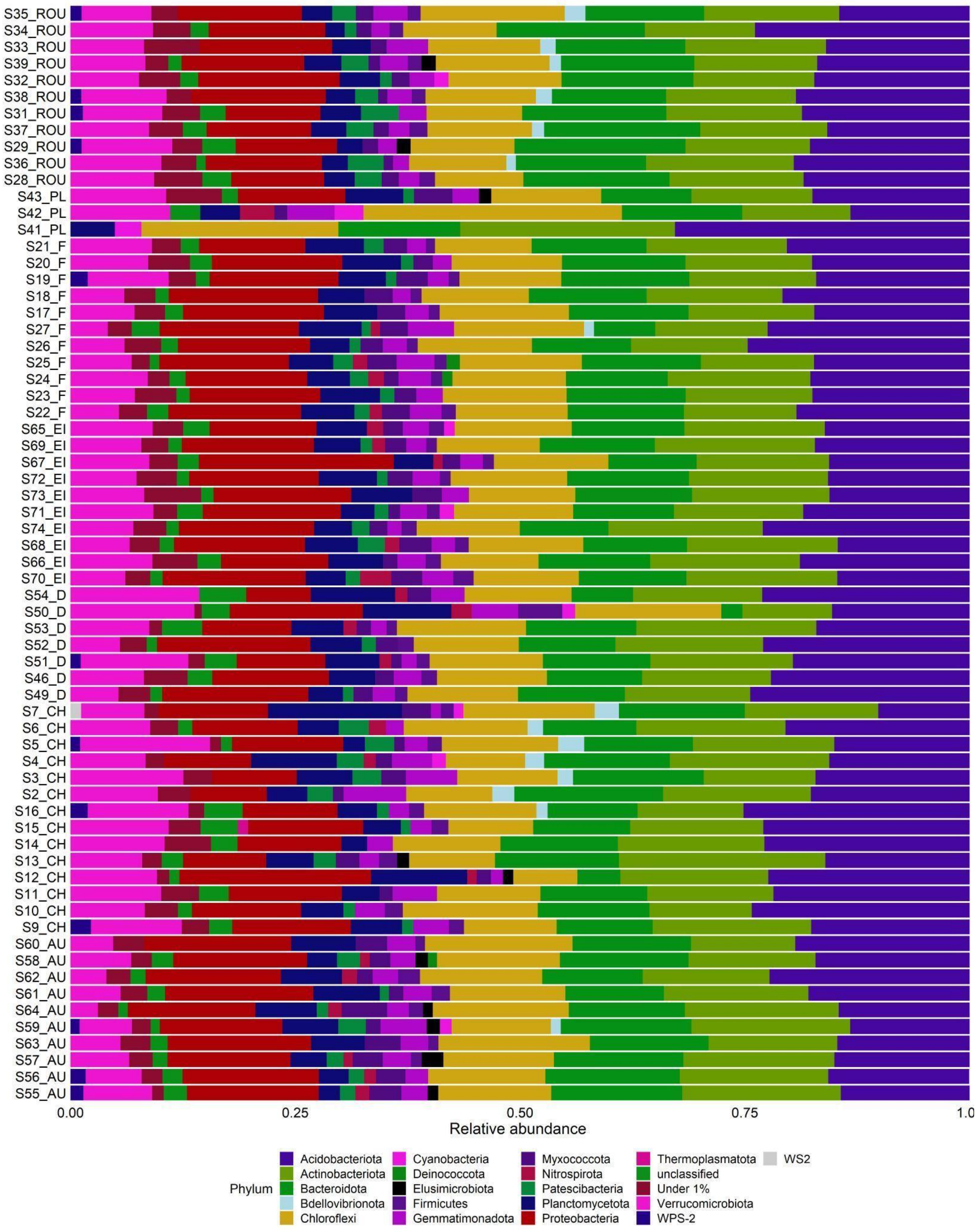

Supplementary Figure 2. Barplot of phylum diversity in all soil sampled across 7 countries. For each sample, the phyla with less than 1% relative abundance were clustered as "Under 1%" to help with visualization.

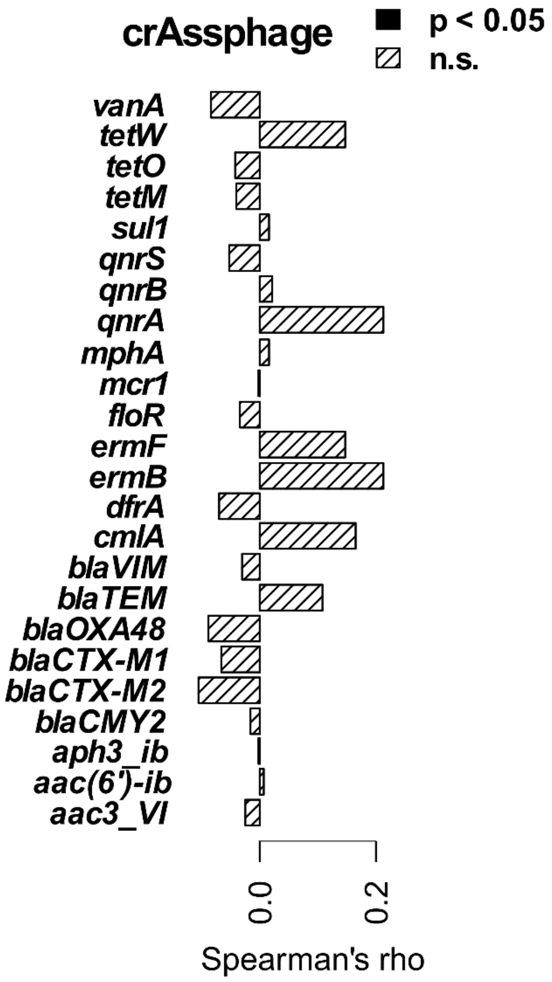

Supplementary Figure 3. Correlation analysis of ARG abundance with observed crAssphage abundance. Spearman rank correlation with Bonferroni correction for multiple testing from river environmental samples are displayed. For soil samples no correlation was possible as crAssphage was not observed in any of the samples. Filled bars represent significant, while hatched bars represent non-significant correlations.

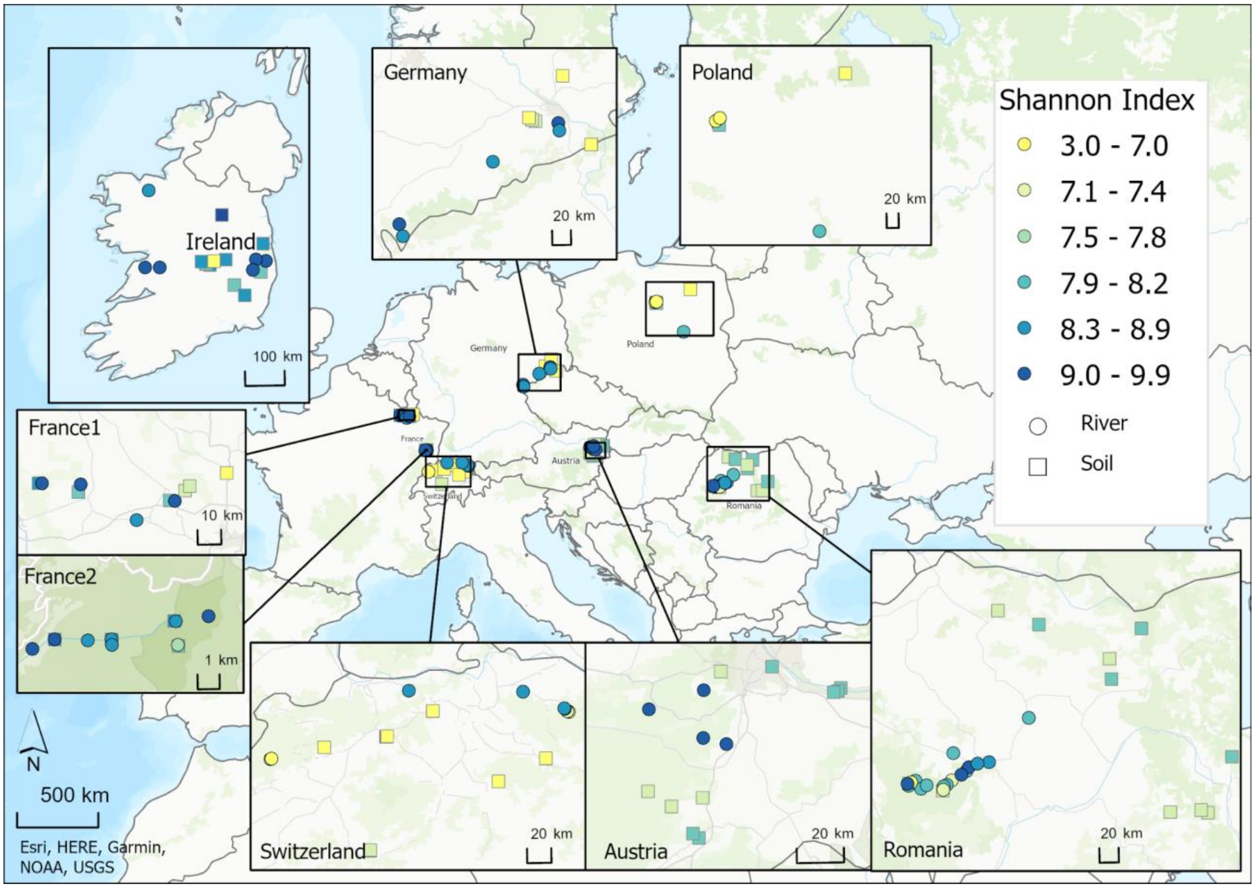

Supplementary Figure 4. Sampling locations across seven European countries. GIS Maps were created in ArcGIS Pro (Esri, Redlands, CA, USA) software using open-source location data. River and regional boundary open-source data was obtained from swisstopo (Swiss Federal Office of Topography) and integrated into the map. River (circle) and soil (square) samples are displayed in the map with their Shannon diversity index indicated through color coding.
