# Supplementary material for: Environmental microbiome diversity and stability is a barrier to antimicrobial resistance gene accumulation
Source: Commun Biol. 2024 Jun 8;7:706. doi: 10.1038/s42003-024-06338-8 (PMC11162449; doi:10.1038/s42003-024-06338-8)
Supplement: Supplementary file 3 — Description of Additional Supplementary Files [file 42003_2024_6338_MOESM3_ESM.pdf]

Description of Additional Supplementary Files

File name: Supplementary data 1
Description: ASV table of river samples and correlation with ARG abundance.

File name: Supplementary data 2
Description: ASV table of soil samples and correlation with ARG abundance.

File name: Supplementary data 3
Description: Soil sample dataset.

File name: Supplementary data 4
Description: River sample dataset.

File name: Supplementary data 5
Description: List of target genes screened through HT-qPCR.

File name: Supplementary data 6
Description: Pairwise Bray-Curtis dissimilarity of soil samples sorted by total ARGs

File name: Supplementary data 7
Description: Pairwise Bray-Curtis dissimilarity of river samples sorted by total ARGs

.

.
