# Supplementary material for: Environmental microbiome diversity and stability is a barrier to antimicrobial resistance gene accumulation
Source: Commun Biol. 2024 Jun 8;7:706. doi: 10.1038/s42003-024-06338-8 (PMC11162449; doi:10.1038/s42003-024-06338-8)
Supplement: Supplementary file 5 — Reporting Summary [file 42003_2024_6338_MOESM5_ESM.pdf]

nature portfolio

Corresponding author(s): Thomas Ulrich Berendonk

Last updated by author(s): Apr 22, 2024

Reporting Summary

Nature Portfolio wishes to improve the reproducibility of the work that we publish. This form provides structure for consistency and transparency in reporting. For further information on Nature Portfolio policies, see our Editorial Policies and the Editorial Policy Checklist.

Statistics

For all statistical analyses, confirm that the following items are present in the figure legend, table legend, main text, or Methods section.

n/a	Confirmed	
☐	☒	The exact sample size (*n*) for each experimental group/condition, given as a discrete number and unit of measurement
☐	☒	A statement on whether measurements were taken from distinct samples or whether the same sample was measured repeatedly
☐	☒	The statistical test(s) used AND whether they are one- or two-sided<br>*Only common tests should be described solely by name; describe more complex techniques in the Methods section.*
☐	☒	A description of all covariates tested
☐	☒	A description of any assumptions or corrections, such as tests of normality and adjustment for multiple comparisons
☐	☒	A full description of the statistical parameters including central tendency (e.g. means) or other basic estimates (e.g. regression coefficient) AND variation (e.g. standard deviation) or associated estimates of uncertainty (e.g. confidence intervals)
☐	☒	For null hypothesis testing, the test statistic (e.g. *F*, *t*, *r*) with confidence intervals, effect sizes, degrees of freedom and *P* value noted<br>*Give P values as exact values whenever suitable.*
☒	☐	For Bayesian analysis, information on the choice of priors and Markov chain Monte Carlo settings
☒	☐	For hierarchical and complex designs, identification of the appropriate level for tests and full reporting of outcomes
☐	☒	Estimates of effect sizes (e.g. Cohen's *d*, Pearson's *r*), indicating how they were calculated

Our web collection on statistics for biologists contains articles on many of the points above.

Software and code

Policy information about availability of computer code

Data collection	N/A
Data analysis	N/A

For manuscripts utilizing custom algorithms or software that are central to the research but not yet described in published literature, software must be made available to editors and reviewers. We strongly encourage code deposition in a community repository (e.g. GitHub). See the Nature Portfolio guidelines for submitting code & software for further information.

Data

Policy information about availability of data

All manuscripts must include a data availability statement. This statement should provide the following information, where applicable:

- Accession codes, unique identifiers, or web links for publicly available datasets
- A description of any restrictions on data availability
- For clinical datasets or third party data, please ensure that the statement adheres to our policy

The datasets supporting the conclusions of this article are included within the article and its additional files. Original sequencing data is available in the NCBI sequencing read archive under project accession number PRJNA948643 (https://www.ncbi.nlm.nih.gov/bioproject/PRJNA948643), with individual sample identifiers given in Tables S3 & S4. Any additional information regarding the manuscript is available through the corresponding author upon reasonable request.

Human research participants

Policy information about studies involving human research participants and Sex and Gender in Research.

Reporting on sex and gender	N/A
Population characteristics	N/A
Recruitment	N/A
Ethics oversight	N/A

Note that full information on the approval of the study protocol must also be provided in the manuscript.

Field-specific reporting

Please select the one below that is the best fit for your research. If you are not sure, read the appropriate sections before making your selection.

☐ Life sciences ☐ Behavioural & social sciences ☒ Ecological, evolutionary & environmental sciences

For a reference copy of the document with all sections, see nature.com/documents/nr-reporting-summary-flat.pdf

Ecological, evolutionary & environmental sciences study design

All studies must disclose on these points even when the disclosure is negative.

Study description

In 7 European countries each, at least 10 independent biofilm/sediment samples from rivers and at least 10 independent forrest soil samples of low anthropogenic impact were taken and analyzed for microbial community composition as well as abundance of 17 antibiotic resistance genes, 5 mobile genetic elements and total bacterial as well as total crAssphage abundance.

Research sample

Low anthropogenic impact samples were selected to avoid any impact of recent pollution events on the resistome.
River biofilm/sediments were collected to obtain environmental samples from a more dynamic, regularly mixed environment.
Soil samples were collected to obtain environmental samples from a more structured environment.

Sampling strategy

Soil sampling and processing
The terrestrial campaign consisted of collecting 74 forest soil samples from the seven countries during fall 2020. The aim was to obtain sample sets that that are of relatively low anthropogenic impact. From each forest location five single core samples (Pürckhauer drill, Buerkle™, Germany) were extracted from a depth of 0-25 cm along two 10 m virtual diagonals laid across the sampling location in the form of an X-pattern. 200 g of each of these five subsamples were combined in an aseptic plastic bag, thoroughly homogenized and transferred to the laboratory at 10 °C. From the composite sample aliquots of 20 g were sieved (2 mm mesh size) and stored at -20 °C. DNA extraction was performed using the DNeasy PowerSoil Pro Kit (Qiagen, Germany) according to the manufacturer's instructions. To obtain DNA from a total of 1 g of each sieved soil sample four replicates of 0.25 g each were extracted in parallel and combined thereafter. At least one extraction blank per country was used to confirm the absence of DNA contamination. The quality and quantity of the extracted DNA was assessed spectrophotometrically.

Riverbed material sampling and processing
The aquatic campaign included the collection of 98 river samples from seven countries (Austria, France, Germany, Ireland, Poland, Romania, and Switzerland) during the fall/winter 2020/21. The locations were selected to obtain samples that are of relatively low anthropogenic impact (e.g., no upstream wastewater treatment plant discharges; no known upstream discharge through agricultural activities or septic systems; no discharge through human recreational areas in the immediate proximity). At each site, the substrate best representing sessile, non-phototrophic, oxygenated microbial communities in the chosen riverbeds, was identified through visual inspection and subsequently sampled. Either epilithic biofilms from the undersides of rocks to avoid phototrophic communities for those streams dominated by rock/gravel, or oxygenated sediment for streams dominated by fine sediment were collected. Specifically, for epilithic biofilm samples, five individual rocks, collected from a shaded sample area from a riverbed length of approximately 10 meters, were gently scraped from the bottom surface using a sterile toothbrush, and combined to create a composite river biofilm sample. Repeated rinsing with sterile water in a 50 mL falcon tube was performed to collect the biomass. If no rocks or rock biofilms were available, fine surface sediment from shaded areas was sampled. In this case, the upper layer (~ 5 cm) of sediment was collected using a 50 mL falcon tube. Five sediment cores were combined at equal weight to obtain one composite sample.
All collected samples were gently homogenized and transported to the laboratory on ice. Then, samples were centrifuged (4,000 rpm for 5 min at 4 °C), the supernatant removed, pellets weighted and stored at -20 °C. DNA extraction was performed using the DNeasy PowerSoil Pro Kit (Qiagen, Germany) according to the manufacturer's instructions. At least one field blank of sterile water mixed with a sterile toothbrush used for obtaining of biofilms and one extraction blank per country were used to confirm the absence of DNA contamination. The quality and quantity of extracted DNA was assessed spectrophotometrically.

Data collection

Amplicon sequencing and analyses of sequence datasets
To analyze the bacterial diversity and taxonomic composition of the samples, DNA extracts were sent to the IKMB sequencing facility (minimum 10,000 reads per sample; Kiel University, Germany). Illumina MiSeq amplicon sequencing of the bacterial 16S rRNA gene

was performed using primers targeting the V3-V4 region (V3F: 5′-CCTACGGGAGGCAGCAG-3′ V4R: 5′-GGACTACHVGGGTWTCTAAT-3) .

High-throughput qPCR of ARGs and genetic markers for MGEs
To determine the relative abundance of target genes in each sample, DNA extracts were sent to Resistomap Oy (Helsinki, Finland) for HT-qPCR analysis using a SmartChip Real-time PCR system (TaKaRa Bio, Japan). The target genes included 27 ARGs and 5 MGEs. In addition, the 16S rRNA gene and the anthropogenic fecal pollution indicator crAssphage were quantified. All samples were run with three technical replicates. The protocol was as follows: PCR reaction mixture (100 nL) was prepared using SmartChip TB Green Gene Expression Master Mix (TaKara Bio, Japan), nuclease-free PCR-grade water, 300 nM of each primer, and 2 ng/µL DNA template. After initial denaturation at 95 °C for 10 min, PCR comprised 40 cycles of 95 °C for 30 s and 60 °C for 30 s, followed by melting curve analysis for each primer set. A cycle threshold (CT) of 31 was selected as the detection limit. The quantification limit was calculated as 25 gene copies per reaction accounting for 12.5 gene copies per ng of DNA template.

Timing and spatial scale: The spatial scale of sample collection spans 7 European countries and the exact GPS coordinates are given in the Supplementary material of the manuscript. Similarly the dates of sample collection are given.

Data exclusions: Samples for which either the 16S sequencing or the high-throughput qPCR analysis failed were excluded from the study as no correlation analysis between diversity and resistance gene abundance was possible.

Reproducibility: Sequencing of Mock Communities and DNA extraction blanks as well as HT-qPCR analysis of DNA extraction blanks were performed. As no experiments were carried out, no experimental repeats were necessary.

Randomization: N/A

Blinding: N/A

Did the study involve field work? ☒ Yes ☐ No

Field work, collection and transport

Field conditions: Please refer to the supplementary material, where the information is presented for the over 150 locations.

Location: Please refer to the supplementary material, where the information is presented for the over 150 locations.

Access & import/export: Only openly accessible locations were chosen, so that no additional permits were necessary.

Disturbance: N/A

Reporting for specific materials, systems and methods

We require information from authors about some types of materials, experimental systems and methods used in many studies. Here, indicate whether each material, system or method listed is relevant to your study. If you are not sure if a list item applies to your research, read the appropriate section before selecting a response.

Materials & experimental systems

n/a	Involved in the study	
☒	☐	Antibodies
☒	☐	Eukaryotic cell lines
☒	☐	Palaeontology and archaeology
☒	☐	Animals and other organisms
☒	☐	Clinical data
☒	☐	Dual use research of concern

Methods

n/a	Involved in the study	
☒	☐	ChIP-seq
☒	☐	Flow cytometry
☒	☐	MRI-based neuroimaging
